# A Novel Endophytic *Trichoderma longibrachiatum* WKA55 With Biologically Active Metabolites for Promoting Germination and Reducing Mycotoxinogenic Fungi of Peanut

**DOI:** 10.3389/fmicb.2022.772417

**Published:** 2022-03-10

**Authors:** Abdulaziz A. Al-Askar, Ehsan M. Rashad, Zeiad Moussa, Khalid M. Ghoneem, Ashraf A. Mostafa, Fatimah O. Al-Otibi, Amr Abker Arishi, WesamEldin I. A. Saber

**Affiliations:** ^1^Department of Botany and Microbiology, Faculty of Science, King Saud University, Riyadh, Saudi Arabia; ^2^Department of Seed Pathology Research, Plant Pathology Research Institute, Agricultural Research Center, Giza, Egypt; ^3^Microbial Activity Unit, Department of Microbiology, Soils, Water and Environment Research Institute, Agricultural Research Center, Giza, Egypt; ^4^School of Molecular Sciences, The University of Western Australia, Perth, WA, Australia

**Keywords:** citric acid, hydrolytic enzymes, biocontrol, biomass, fermentation, response surface methodology, peanut, bioactive metabolites

## Abstract

Plant residuals comprise the natural habitat of the plant pathogen; therefore, attention is currently focusing on biological-based bioprocessing of biomass residuals into benefit substances. The current study focused on the biodegradation of peanut plant residual (PNR) into citric acid (CA) through a mathematical modeling strategy. Novel endophytic *Trichoderma longibrachiatum* WKA55 (GenBank accession number: MZ014020.1), having lytic (cellulase, protease, and polygalacturonase) activity, and tricalcium phosphate (TCP) solubilization ability were isolated from peanut seeds and used during the fermentation process. As reported by HPLC, the maximum CA (5505.1 μg/g PNR) was obtained after 9 days in the presence of 15.49 mg TCP, and 15.68 mg glucose. GC–MS analysis showed other bioactive metabolites in the filtrate of the fermented PNR. Practically, the crude product (40%) fully inhibited (100%) the growth and spore germination of three mycotoxinogenic fungi. On peanuts, it improved the seed germination (91%), seedling features, and vigor index (70.45%) with a reduction of abnormal seedlings (9.33%). The current study presents the fundamentals for large-scale production in the industry for the sustainable development of PNR biomass as a natural source of bioactive metabolites, and safe consumption of lignocellulosic-proteinaceous biomass, as well. *T. longibrachiatum* WKA55 was also introduced as a novel CA producer specified on PNR. Application of the resulting metabolite is encouraged on a large scale.

## Introduction

The agricultural proteinaceous-lignocellulosic residues (PNR) of the above-ground part of groundnut or peanut (*Arachis hypogaea* L.) represent an environmental issue if they are not wisely managed. Moreover, they can be biologically converted into valuable biomolecules. On the dry matter (93.39%) base, ash composed 5.33%, and the organic matter; 94.67%, the latter has a high level of protein (8.08%), and the remaining mostly composed of cellulose, hemicellulose, lignin, and pectin, and thus, it been used as a ruminant feedstuff (Alebel et al., 2019). The degradation of cellulose, hemicellulose, lignin, pectin, and protein polymers requires the catalytic action of cellulases pectinase and proteases enzymes to yield various corresponding monomers, such as mono-sugars and D-galacturonic acid as carbon and energy source, whereas the proteolytic activity is referred to the protease enzymes that yield various amino acids, which perform a critical role in the growth and multiplication of the microorganisms (Jacob et al., 2008; Elsayed et al., 2021; Al-Askar et al., 2021a).

Experiments in recent decades have produced a large quantity of evidence on the prospective use of helpful endophytic microorganisms to manage diseases. Endophytic microbiome (endosymbionts) is defined as the diverse array of microbial communities that live and grow intra-and/or intercellularly in the plant’s tissues, at least part of its life cycle, without causing over symptoms on the host plants. Endophytes may benefit hosts through a variety of mechanisms, including molecules that increase their ability to compete for space, nutrients, and/or ecological niches, as well as the synthesis of antimicrobial substances, phytohormones, and peptides that may keep vegetables and plant organs healthy. Fortunately, all these substances have no negative impact neither on consumers nor on the environment (Shehata et al., 2016; López et al., 2018).

Of the seed-borne endophytes, the fungal seed microbiome is an important variable branch that may be deeply impacted by local conditions and host genotypes (Klaedtke et al., 2016). Although fungal seed-borne endophytes comprise a significant part of the seed microbiome, to date, this fungal microbiome has not been fully explored. Superior to other fungi, endophytic *Trichoderma* spp. produce a novel array of metabolites that exhibit different bioactivities with diverse applications in pharmaceutical, industrial, plant growth regulation, and many others (De Silva et al., 2019; Sridharan et al., 2020, 2021). Therefore, this study is a trial to shed some light and share some information about such a limited-explored area.

Despite being an inconvenient task, finding out a suitable microorganism having the complete and complementary catalytic action to manage the biodegradation of PNR is of great importance. Proper bioprocessing of PNR is a very beneficial and talented approach and potentially offers a low-cost raw resource to various valuable bioproducts. Alternative to the conventional substrates, proteinaceous-lignocellulosic materials such as PNR are abundant, attractive, and usually low-priced as well as, provide the required nutrients to the microorganisms, and are suitable substrates for their growth and activity (Kumari et al., 2008; Max et al., 2010).

Most of the previous work on plant biomass used two-phase bioconversion, i.e., the hydrolysis of complex materials to fermentable monomers, followed by microbial fermentation of monomers to a target product(s). The biodegradation is usually catalyzed by hydrolytic enzymes and the fermentation process is run by a proper microbe (Saber et al., 2015; Al-Askar et al., 2021b).

Solid-state fermentation (SSF) was applied during the bioconversion process. What is nominated as the best choice of fermentation of plant biomass is the low wastewater output, minimum production cost, fewer energy requirements, high rate of efficiencies, easier aeration, and simple fermentation medium (Show et al., 2015). These advantages could be maximized when using proper experimental design, of which central composite design (CCD) of response surface methodology (RSM) is a precise and easily applicable statistical approach for building a mathematical empirical model for maximization of a target product, in contrast to conventional procedures; this design is used to identify the importance and interactions of the input factors (independent variables) and select conditions that optimize the output response (variable) using a limited number of experiments (runs) even when little is known about the bioprocess conditions (Saber et al., 2015; Asadi and Zilouei, 2017).

The importance of the current study arises from the point of view that much remains to be studied for developing a commercially feasible process for the utilization of alternative disposal of PNR. Also, of the points to be improved is to find out alternative efficient fungus in bioconversion of PNR directly into the target biopolymer. The current work was set up to merge the two-phase bioconversion of PNR in one single step, in which the resulting monomers from the biodegradation process can share directly to the formation of the target molecules, e.g., citric acid (CA) (2-hydroxy-propane-1,2,3-tricarboxylic acid).

Herein, a novel endophytic isolate of *Trichoderma* sp. was reported as a biologically active fungus capable to produce CA, as a value-added product by the bioprocessing of PNR biomass under a one-phase conversion process, applying SSF technique and CCD. The fungal exudate was evaluated as a suppressor to the associated mycotoxinogenic and pathogenic fungi that settled in biomass and peanut seeds as well.

## Materials and Methods

Unless otherwise specified, all chemicals used through the enzymatic and biochemical analysis are of a high grade obtained from Sigma-Aldrich Company (Egypt brach).

### Isolation of Endophytic Fungi of Peanut

Fifteen-seed samples of peanut plants were collected from different locations in Al Nubaria district, and AL-Behaira and Sohage governorates. The surveyed area lays between latitudes of 26°49′N and 31°11′N, and longitudes of 29°68′E and 31°73′E, Egypt, during June 2020. The samples were gathered from an area of 50 m× 50 m around each sampling site in a random zigzag pattern. Full mature peanut pods were gathered in cotton bags, labeled in the field, and stored at 4°C until the isolation process.

The discovery of endophytic seed-borne fungi was performed, utilizing the agar plate technique recommended by ISTA (2007). A total of 200 healthy-looking seeds of each sample were surface sterilization, applying ethanol (70% for 60 s), then by sodium hypochlorite (2% for 90 s), and again in ethanol (100% for 30 s); finally, the seeds were washed using sterilized water for five times and dried using sterilized filter papers under laminar air flow chamber. For each plate, 10 were plated on a potato dextrose agar (PDA) plate (Difco, United States). To prevent any bacterial growth, L-chloramphenicol and streptomycin sulfate (5 mg/L each) were added to the medium. The seed-containing dishes were reared at 20 ± 2°C under cool white fluorescent light with alternating cycles of 12 h light/darkness.

Along 7 days, plates were inspected under stereoscopic and compound microscopes to pick the emerged fungi. Hyphal-tip and/or single-spore isolation procedures were used to gain pure cultures of endophytic fungi. The percentage of occurrence of each isolated endophyte fungal species was calculated using equation (10) follows:


(1) $$\mathrm{Mean percentage of}\;a\;\mathrm{fungus}=\frac{\left(\Sigma \;\mathrm{fungus percentage in examined samples}\right)\;}{\mathrm{Total number of examined samples}\;}$$

All endophyte fungi were maintained on slants of PDA for further investigation. Fungi were identified according to their cultural features, fungal morphology, and microscopic characteristics (Ellis, 1971, 1976; Domsch et al., 1980; Nelson et al., 1983; Leslie and Summerell, 2008; Samson et al., 2014).

### Screening the Lytic Activity of the Endophytic Fungi

The isolated endophytic fungi were first descriptively screened for cellulolytic activity by growing on plates of agar medium containing 0.5% carboxymethyl cellulose (CMC) at 25°C and examined daily up to 5 days. Active cellulolytic isolates were determined by the formation of a clear zone after flooding with 0.2% Congo red for 15 min, then de-stained by washing twice with 1 M NaCl for 15 min (Meddeb-Mouelhi et al., 2014). Following the previous primary screening, seven isolates were selected, and quantitatively screened for cellulase, and protease activities, as well as phosphate solubilization ability by growing on a fermentation medium based on PNR.

#### Preparation of PNR

The whole PNR was collected from the regions reported above, dried at 50°C overnight, and blinded in an electric grinder to get fine residue (1 mm in length) to serve as solid sustenance and substrate for during fermentation production. PNR was a natural growth substrate to simulate the natural growth situations of the fungi, and therefore, was not subjected to any other pretreatment.

#### Fermentation Procedure

The SSF technique was used for quantitatively screening the capability of the selected seven isolates to degrade PNR into a simpler form, as well as the solubilization of complex tricalcium phosphate (TCP). Unless otherwise specified, 1 g of PNR was mixed thoroughly with 20 mg TCP, and 5 ml tap water, pH 5, in 250 ml conical flasks, the PNR-based fermentation medium was autoclaved for 15 min at 121°C. After sterilization, 1 ml of spore suspension, containing 108 spores/ml, from each fungus was used to inoculate the fermentation media. During the incubation period, the moisture content was retained at approximately 65%. Unless otherwise specified, the fermentation lasted for 7 days at 28°C. By the end of the incubation period, 10 ml of 0.01% Tween 80 was integrated with the fermented matter and kept on a rotary shaker (150 rpm for 30 min), then filtered through Whatman No. 1 filter paper before being centrifuged at 5,000 rpm for 20 min. The residue of PNR after SSF was oven-dried (50°C) to constant weight for estimation of reduction in PNR weight after fermentation. The post-culture filtrate (PNR hydrolysate) was biochemically evaluated. The time course profile of PNR bioconversion into organic acids was determined.

#### Biochemical Analysis

Cellulases in the post-culture filtrate was carried out following the protocols of Elsayed et al. (2021) with slight modification, in which the activities of filter-paperase (FPase), carboxymethyl cellulase (CMCase), and hemicellulose-degrading xylanase on 1% of microcrystalline cellulose, carboxymethyl cellulose, and oat-spelt xylan were assayed, respectively. All substrates were individually suspended in citrate buffer (0.05 M, pH 4.8), the reaction blend (1 ml of the filtrate and 1 ml appropriate substrate-buffer solution) was incubated at 50°C for 60, 30, and 15 min, respectively. The resulting free reducing sugars by the enzymatic action was detected using the 3,5-dinitrosalicylic acid method (Miller, 1959), and the developed color was measured using UV-VIS spectrophotometer (Jenway 7,315) at wave length A_575_ nm. Enzyme unit (U) is defined as the amount of enzyme required to release one μmol min^−1^ of glucose (FPase or CMCase) or xylose (xylanase) per g PNR/min under the assay conditions.

Polygalacturonases (PGase) was assayed by detecting the reducing monomers released from 1% polygalacturonic acid in 0.1 M sodium acetate buffer (pH 5.2) after 30 min incubation at 40°C (Al-Askar et al., 2021a), using the 3,5-dinitrosalicylic acid method as described above with D-galacturonic acid monohydrate as the standard. One PGase unit was described as that amount of enzyme that yields 1 μmole reducing ends per g PNR/min under the assay conditions.

The proteolytic activity (protease) was quantitatively assayed in the crude extract using 2.5 ml casein (1%) as a substrate incubated with 1 ml crude enzyme, then incubation at 37°C for 10 min, and 2.5 ml TCAA was added. One milliliter of the supernatant was used for detection of the resulted-free amino acids spectrophotometrically at A_660_ nm (Negm et al., 2021). One protease U was termed as the amount of the enzyme that releases 1 μg of tyrosine equivalent per g PNR/min under the assay settings.

The soluble phosphorus was determined in an aliquot of the sample (5 ml) was pipetted into a 25 ml volumetric flask; then, NH_4_-molybdate solution (5 ml) was slowly added then shaken gently. To which, 1 ml sodium sulfite (20%) was added. The mixture was shaken and 1 ml hydroquinone (0.5%) was added and then was shaken. Total phosphorus was determined spectrophotometrically A_660_ nm after 15 min (Jackson, 2005).

The total organic acids were also determined (Montgomery et al., 1962). A combination of 0.5 ml of the sample, 1.5 ml of ethylene glycol, and 0.2 ml of diluted sulfuric acid were mixed, followed by heating in a boiling water bath for 3 min, then immediately cooling in cold water. To the mixture, 0.5 ml of hydroxyl ammonium sulfate solution and 2.0 ml of 4.5 N sodium hydroxide were added. Then, 10 ml of acidic ferric chloride was added. The optical density was measured at A_500_ nm within 1 h of color development.

The post-culture filtrate was analyzed for glucose content utilizing the kit of glucose oxidase (Spainreact Co., Spain). The pH of the post-culture filtrate was measured by a glass electrode pH-meter (CP-501, Elmetron). The dry weight of the residual fermented PNR was measured by drying at 50°C until a constant weight was attained.

### The Matrix of CCD Used for Maximization of CA Biosynthesis

For bioprocessing the maximization of CA biosynthesis by *Trichoderma* sp. WKA55, the SSF of PNR-based medium was applied, using the experimental matrix of CCD. In which one-gram PNR was mixed with two independent nutritional variables (glucose and TCP) at five concentrations each. According to the design matrix of CCD, five coded levels from each variable were allocated at the center (0), low (−1), high (1), and two axial levels (alpha, *α* ± 1.414), the corresponding uncoded (actual) points were 10, 20, 15, 7.929, and 22.071 mg TCP per g PNR of both glucose and TCP. A total of 11 combinations of the medium (run) were prepared, autoclaved, and inoculated with the spore suspension (108/ml per g PNR) of the fungus. After incubation (28°C), CA was quantified by high-performance liquid chromatography (HPLC). The next second-order polynomial quadratic model (Equation 2) is used for theoretical valuation of the optimum level of each variable that maximizes CA biosynthesis:


(2) $$Y=\beta_{0}+\sum \beta_{i}X_{i}+\sum \beta_{ij}X_{i}X_{j}+\beta_{ii}X_{i}^{2}$$

Where, *Ү*, the predicted CA; *β_0_*, the model coefficient; *β_i_*, the linear coefficients; *β_ij_*, the interaction coefficients; *β_ii_*, quadratic coefficients; and *X_i_*, and *X_j_*, the independent variables of glucose and TCP, respectively.

### Determination of CA by HPLC

Methanol (20 ml) was used for extraction of CA from the sample at 40°C, then filtering and concentrating under reduced pressure until dryness, followed by redissolving in acidified water (pH 2) with HCl, then evaporated at 40°C under dryness, and reliquefied again in 1 ml H_2_SO_4_ (0.01 N). For the CA examination, 20 μl was injected in HPLC (Agilent 1,200 Infinity Series, United Kingdom), with a C18 column at 30°C. Elution was carried out isocratically with H_2_SO_4_ (0.01 N), as the mobile phase, at a flow rate of 0.1 ml/min, for 120 min. The UV detection was accomplished at 214 nm. CA was quantified by the absorbance verified in the chromatograms relative to the CA standard (Silva et al., 2002).

### Evaluation of Crude CA

#### *In vitro* Assessment of Crude CA on Mycotoxinogenic Fungi

The toxigenic fungi: *Aspergillus flavus* PPRI3, *Aspergillus ochraceus* PPRI5, and *Fusarium oxysporum* PPRI10 were kindly provided by Seed Pathology Research Department, Plant Pathology Research Institute, Agricultural Research Center (ID: 60019332), Giza, Egypt. These fungal strains are toxigenic and are reported by the provider to produce aflatoxin, ochratoxin, and fusaric acid, respectively. CA was produced under the optimized fermentation conditions of PNR with TCP and glucose after incubation at 28°C for 9 days as the optimum fermentation period. The resulting crude CA was sterilized by microfiltration using membrane filters with 0.45 μ pore size.

The minimum antifungal concentration (MIC) of the crude CA of *Trichoderma* sp. WKA55 was evaluated against the three toxigenic fungi. One hundred milliliters Erlenmeyer flasks containing 20 ml sterilized Czapek-Dox broth medium (Sigma-Aldrich) with various concentrations of CA (0%, 10%, 20%, 30%, and 40%, v/v) were inoculated with 0.2 ml of 10^6^ conidia/ml of the tested fungal spore suspensions prepared from 6-day-old cultures, then incubated at 28°C for 6 days. At the end of the incubation period, the mycelial mats were harvested, washed with distilled water, and oven-dried at 50°C to constant weight for measuring the growth of tested fungi. The reduction in the growth of mycotoxinogenic fungi was calculated according to the following Equation (3):


(3) $$\mathrm{Growth}\;\mathrm{reduction}\;\left(\%\right)=C-T/C\times 100$$

Where *C* is the average mycelial dry weight of each pathogen in the control and *T* is the average dry weight of each pathogen in the crude CA treatment.

The anti-activity of the crude CA on germination of the fungal conidia was assayed in test tubes containing 10 ml sterilized Czapek-Dox broth medium supplemented with the same previous concentrations of *CA*. All tubes were incubated individually with 0.2 ml of 10^6^ conidia/ml of the spore suspensions, then incubated at 28°C for 12 h. With the aid of a light microscope, the percentage of spore germination was then counted using a hemocytometer slide (Elad, 1992).

#### CA Against Seed-Borne Fungi and Seed Germination

The influence of CA on the occurrence of seed-borne pathogenic fungi, germination, and vigor index of peanut seeds (Giza 5), a common susceptible greenhouse cultivar to Fusarium wilt in Egypt, was investigated. Three seed lots, with the lowest germination (~70%), having high infection by the previously tested; *A. flavus*, *A. ochraceus*, and *F. oxysporum* phytopathogens were selected.

The seeds of each sample were individually soaked in the bio-prepared CA for 30 min, at the best concentration obtained from the preceding MIC test, and the seeds were dried for 2 h under a sterile air stream (Singh et al., 2016). Seeds that were soaked in sterile distilled water were served as control. The seeds were incubated (20 ± 2°C) and relative humidity of 50% for 14 days with rotating cycles of light and darkness every 12-h, in a germination moist chamber. The germination test was conducted on 400 seeds using the standard moist blotter technique (ISTA, 2007). After 7 days of incubation, the fungal community that developed on the seeds was screened with the use of a stereoscopic microscope and confirmed with the optical microscope. The results were expressed as the percentage per pathogen detected.

Germinated seeds were counted at 7 and 14 days and results were expressed as germination percentage. Seed with a radical length of five millimeters or more was counted as germinated. For vigor evaluation, the root length of 100 seeds was measured at 7 and 14 days after planting. Seedling’s vigor index (SVI) was calculated with the average root length and germination percentage of each seed sample. The vigor index was assessed utilizing the following Equation (4; Abdul-Baki and Anderson, 1973).


(4) $$SVI=\mathrm{Germination}\;\mathrm{percentage}\;\left(\%\right)\times \mathrm{average}\;\mathrm{root}\;\mathrm{length}\;\left(\mathrm{cm}\right)$$

Other disinfected seeds were treated with a recommended fungicide [Pink-S (5-methylisoxazol-3-ol) at 30% SL] for 15 min and used as a positive control. A set of untreated seeds was served as a negative control. The germination (%) and the measurement of the root lengths (cm), fresh and dry weight (g) were estimated on the 14th day.

### Gas Chromatography-Mass Spectrophotometry

The investigation of GC-MS of the cell-free fungal filtrate was carried out, using Agilent 6,890 gas chromatography prepared with an Agilent mass spectrometric detector, with a direct capillary interface and fused silica capillary column PAS-5 ms (30 mm × 0.25 μm film thickness) (GC-MS-QP 2010 Shimadzu, Japan). The carrier gas (helium) was used at approximately 1 ml/min, pulsed split less mode. The solvent delay (3 min) and the injection size (1.0 ml) were adjusted. The mass spectrophotometric detector was functioned in electron impact ionization mode in ion energy of 70 eV, scanning from *m/z* 50 to 500. The temperatures ion source and quadrupole were adjusted at 230°C and 150°C, respectively. The electron multiplier voltage was kept 1,250 v above autotune. The instrument was manually tuned by perfluorotributylamine. The temperature program of GC was started at 60°C for 2 min then elevated to 280°C at a rate of 5°C/min, and 10 min hold at 280°C, the injector temperature was set at 280°C. Separated peaks were recognized using NIST08s.LIB mass spectral database (Sparkman et al., 2011).

### Fungal Identification on Molecular Base

The selected fungus was molecularly identified. Frozen-dried mycelium (20 mg) was crushed with Kontes pestles in a 1.5 ml tube with 500 μl of Dellaporta buffer (100 mM Tris pH 8. 50 mM ethylenediamine-tetraacetate EDTA, 500 mM NaCl, and 10 mM beta-mercaptoethanol) in liquid nitrogen; then, 33 μl of sodium dodecyl sulfate (20%, w/v) was added, the mixture was vortexed and incubated (65°C) for 10 min. Potassium acetate (160 μl of 5 M) was added and vortexed. Then, spun for 10 min at 10,000 rpm in a microcentrifuge tube. The supernatant (450 μl) was mixed with 450 μl phenol, chloroform, and isoamyl-alcohol (25:24:1) and vortexed for 5 min. Then, spun for 5 min at 10,000 rpm. Four hundred microliter of the upper phase was moved to a clean microcentrifuge tube and mixed with 0.5 volumes of isopropanol, vortex, and spun (10 min at 14,000 rpm). The supernatant was detached, and the precipitated nucleic acid was washed with 70% ethanol, spun (5 min at 10,000 rpm), and finally resuspended in 100 μl of ddH_2_O.

Amplification was carried out *via* PCR based on 18 s rRNA primers and sequencing of the internal transcribed spacer (ITS) region (White et al., 1990). The forward IT5 primer (5′-GGAAGTAAAAGTCGTAACAAGG-3′) and the reverse ITS4 primer (5′-TCCTCCGCTTATTGATATGC-3′) were utilized to magnify the entire ITS region. PCR was done in a reaction, containing 1 μl of the fungal DNA extract (40 ng of total DNA), 2 mM MgCl_2_, 2.5 of 10x PCR buffer, 1.5 μl of 10 μM of each primer, 2.5 μl of 10 mM dNTPs, and 0.3 μl of 5 U Taq DNA polymerase and the reaction was completed to 25 μl with Nuclease-free water. PCR was conducted in the ESCO Swift Maxi Thermal Cycler with initial denaturation at 95°C for 2 min, followed by 35 cycles of 95°C for 30 s, 52°C for 30 s, and 72°C for 30 s, and the final cycle is a polymerization that was made at 72°C for 10 min. PCR products were purified using QIAquick® PCR Purification Kit (Cat. No. 28106) as the manufacturing procedures. The purified PCR products were sequenced (Macrogene Company, Seoul, Korea) in both directions using ITS5 and ITS4 primer pairs. The sequence (613 bp) was aligned and deposited into the GenBank database^1^ to get similarities with the related fungal sequence (nr/nt) database.

A phylogenetic tree was built based on the ITS region sequence comparisons of length polymorphism of the amplified PCR, and sequences from the database using blast tree and aligned using aligned sequences nucleotide BLAST. The phylogeny was tested using the bootstrap method with 1,000 replications and generated based on the Jukes-Cantor model. The software package: MEGA ver. 10 (Newman et al., 2016) was used for multi-alignments and phylogenetic analysis based on the neighbor-joining method. The obtained sequence was deposited in GenBank, and the accession number of the fungal strain was received.

### Statistical Design and Analysis

All data were the mean of at least triplicates. For optimization of CA production, Design-Expert software (version 12, Stat-Ease, Inc. Minneapolis, MN, United States) was used for constructing the CCD matrix and ANOVA of the data. One-way randomized blocks design was used to construct the experimental scheme of both the germination test and fungal load test of peanuts. CoStat software version 6.4 (CoHort Software, United States) was used to perform the one-way ANOVA and verify the difference between the averages based on Tukey’s HSD test. The other data were introduced as mean ± SD. All the statistical hypothesis tests were made at a probability (*P*) value of ≤0.05.

## Results

### Prevalence of Endophytic Fungal in Peanut Seeds

**Figure 1:**
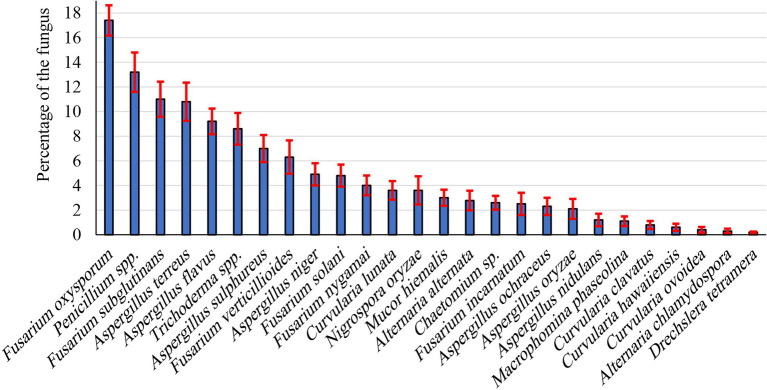
Prevalence of endophytic fungal species isolated from peanut seeds.

During the present investigation, endophytic fungi were isolated from peanut seeds. Most isolates were recorded the first 5 days of incubation. Twenty-six endophytic fungal species belonging to 11 genera were isolated from collected peanut seed samples (Figure 1). Most species belong to Ascomycetes and Deuteromycetes. Of these, the most frequently occurring genera were Fusarium, *Aspergillus*, and *Penicillium*, being 1.20%–10.8%, 2.5%–11.0%, and 13.20%, respectively. *Fusarium* (six species) and Aspergillus (seven species) were the most common genera, representing 23.08% and 26.92% of total fungi, respectively. In this respect, *F. oxysporum* (17.4%) and *F. subglutinans* (11.0%) were the dominant species recovered among all *Fusarium* species, followed by *Fusarium verticillioides* (6.30%), whereas *F. nygamai*, *F. solani*, and *F. incarnatum* were the lowest dominant species. *Penicillium* was the second most prevalent genus of the recovered endophytic fungi. *Aspergillus* spp. ranked third in the frequency in peanut, comprising 26.92% of total fungi. The genus was represented by seven species of which the most dominant species were *Aspergillus terreus*, *A. flavus*, and *Aspergillus sulphureus*, being 10.8%, 9.2%, and 7.0%, respectively, whereas *A. niger*, *A. ochraceus*, *Aspergillus oryzae*, and *Aspergillus nidulans* were the least dominant species. Meanwhile, *Trichoderma* spp. was recorded at moderate percentages (8.60%). Other genera, including *Chaetomium*, *Curvularia*, *Drechslera*, *Macrophomina*, *Mucor*, and *Nigrospora*, were recorded as the lowest endophytes in peanut seeds.

### Cellulolytic Profile of the Endophytic Fungi

**Figure 2:**
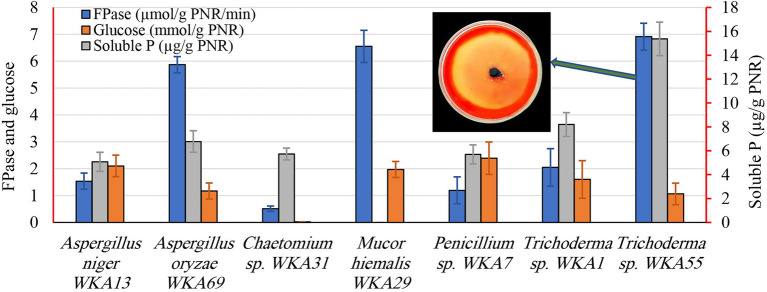
Screening of the endophytic fungi for the cellulase, glucose, and the solubilization of tricalcium phosphate (TCP) after growing on a PNR-based fermentation medium. The figure shows the descriptive assay on the agar plate for detection of cellulolytic activity of *Trichoderma* sp. WKA55.

To perform the bioconversion of PNR into a valuable biomolecule, two sequential screening tests were performed, the first was a descriptive screening experiment, which was conducted to explore the cellulolytic activity of all the isolated endophytic fungi. For the selection of the endophytic fungus that can degrade PNR, the study was initiated by testing the isolated endophytic fungi for their ability to clarify the medium of microcrystalline cellulose agar plates. The ability of a fungus to form a clear zone around the growing colony was considered as a response to the cellulose-degrading ability of the fungus. *Trichoderma* spp. WKA55 exhibited the highest cellulolytic zone around the fungal growth on the agar plate (Figure 2). Accordingly, seven colonies with cellulase activity were obtained. The other isolates were eliminated since they failed to either grow or solubilize cellulose on agar plates. Subsequently, the selected fungi were subjected to further evaluation procedures.

During the second screening test (Figure 2), the seven fungal isolates selected based on the aforementioned screening study were undergone to quantitative screening of FPase activity and the release of glucose on the PNR-based medium, to compare the PNR degrading ability of various fungi. Also, of the fungal solubilization of complex TCP was evaluated, such a process is usually coupled with the synthesis of diverse organic acids. All fungi possess FPase activity but differed greatly in the degree of cellulolytic activity, *Trichoderma* sp. WKA55 (6.914 U), *Mucor* sp. WKA29 (6.554 U), and *Aspergillus* sp. WKA69 (5.872 U) recorded the highest activity. Despite Trichoderma sp. WKA55 was the most active cellulolytic fungus in PNR degradation on both descriptive and quantitative tests, the net accumulation of liberated glucose (1.06 mmol/g PNR) was lower compared with the other fungi.

### Time *vis* Biodegradation of PNR by *Trichoderma* sp. WKA55

The profile of PNR biodegradation against 15 days period by *Trichoderma* sp. WKA55 was explored (Table 1). It looks that the time course sketch of FPase, CMCase, and xylanase during SSF time was similar and these enzymes are synthesized under similar circumstances. The enzymatic system (FPase, CMCase, and xylanase) associated with and involved in the biodegradation process raised continuously from the binging of fermentation up to the 11th day, then decreased markedly with the progress of the incubation period. Data of protease and PGase showed also a reasonable peak of proteolytic (2.746 U) and pectinolytic activity (0.431 U) after 11 days of fermentation. The activity started from the beginning and lasted until the end of incubation (15 days). Moreover, the filtrate of the fermentation PNR biomass showed positive hydrolysis and liberation of soluble P, which reached its maximum (27.46 μg/g PNR) after 11 days of incubation. The data show also that the maximum biosynthesis of the total organic acids (227.12 mmol/g PNR) by *Trichoderma* sp. WKA55 was reached after 9 days of PNR fermentation, this was associated with an obvious reduction in both post-culture filtrate pH and residual PNR biomass by 3.01% and 37.81%, respectively.

**Table 1:** Time course profile of PNR biodegradation for organic acid production in association with cellulolytic activity and TCP solubilization by *Trichoderma* sp. WKA55 grown on a PNR-based fermentation medium.

Time, day	Lytic activity (U/min)					Reduction, %		Soluble P (μg/g PNR)	OA (mmol/g PNR)
	FPase	CMCase	Xylanase	Protease	PGase	pH	Dry weight		
1	1.42 ± 0.49	0.46 ± 0.03	0.20 ± 0.08	0.12 ± 0.03	0.10 ± 0.01	0.30 ± 0.02	1.52 ± 0.31	1.22 ± 0.50	12.0 ± 1.7
3	4.36 ± 0.87	1.10 ± 0.07	2.63 ± 0.89	1.36 ± 0.04	0.21 ± 0.03	1.71 ± 0.27	13.00 ± 0.40	13.57 ± 2.64	75.7 ± 2.6
5	6.12 ± 0.38	2.46 ± 0.31	13.07 ± 2.36	1.44 ± 0.05	0.23 ± 0.09	2.47 ± 0.47	21.18 ± 0.51	14.44 ± 3.41	94.2 ± 2.5
7	6.91 ± 0.20	3.93 ± 0.16	14.96 ± 3.44	1.61 ± 0.13	0.25 ± 0.05	3.15 ± 0.99	29.03 ± 0.53	16.06 ± 3.11	170.5 ± 2.7
9	8.07 ± 0.51	4.96 ± 0.32	42.90 ± 5.59	2.25 ± 0.03	0.35 ± 0.04	3.01 ± 0.20	37.81 ± 1.33	22.51 ± 3.27	227.1 ± 5.3
11	9.07 ± 0.43	6.36 ± 0.51	60.46 ± 5.91	2.75 ± 0.21	0.43 ± 0.05	3.41 ± 0.43	37.96 ± 2.08	27.46 ± 4.01	216.8 ± 4.5
13	7.48 ± 0.43	4.09 ± 0.30	23.89 ± 2.76	2.54 ± 0.23	0.40 ± 0.06	3.02 ± 0.48	38.26 ± 2.38	25.39 ± 3.55	202.6 ± 4.1
15	6.91 ± 0.20	4.05 ± 0.35	13.16 ± 1.46	2.44 ± 0.15	0.38 ± 0.08	2.97 ± 0.52	37.62 ± 1.59	24.78 ± 1.69	198.8 ± 4.8

### CCD for Maximization of CA Biosynthesis

The content of organic acids was differentiated in the post-culture filtrate using HPLC, CA was found to be the major one, signifying about 80% of the detected organic acids. Therefore, the next optimization trial was carried out on CA production on a PNR-based medium under SSF by *Trichoderma* sp. WKA55. The SSF medium of PNR was supplemented with glucose and TCP to support the biosynthesis of *CA*. The incubation lasted for 9 days based on the preceding results. The production of CA was maximized, depending on the full CCD matrix of RSM (Table 2).

**Table 2:** Values of CA levels detected by HPLC based on the central composite design (CCD) matrix of the studied variables (glucose and TCP) on the PNR-based medium fermented by *Trichoderma* sp. WKA55.

Run	Input variables				Output variable
	Coded unit		Actual unit (mg/g PNR)		Citric acid (μg/g PNR ± SD)
	Glucose	TCP	Glucose	TCP	
1	−1	−1	10	10	885.5 ± 3.5
2	−1	1	10	20	966.9 ± 6.4
3	1	−1	20	10	1478.4 ± 28.0
4	1	1	20	20	2887.5 ± 14.4
5	0	−1.414	15	7.929	1322.2 ± 23.1
6	0	1.414	15	22.071	2159.3 ± 20.8
7	−1.414	0	7.929	15	1587.3 ± 11.6
8	1.414	0	22.071	15	2413.4 ± 8.1
9^*^	0	0	15	15	5555.7 ± 12.1
10^*^	0	0	15	15	5357.1 ± 11.7
11^*^	0	0	15	15	5459.3 ± 11.6

* The middle concentrations.

The produced CA in the 11 experimental runs was determined by HPLC, runs 9, 10, and 11 represent the best combination of medium composition for CA production (average = 5457.4 μg CA/g PNR). These were the replicates of three center points of the experimental design for the tested variables (15 mg/g PNR from glucose and TCP). On the other hand, the fermentation conditions of run 1 excreted the lowest amount of CA (885.5 μg CA/g PNR). The highest value of CA situated nearby the center points of the investigated variables reflected the accuracy and fitness of the selected concentrations of each variable.

#### ANOVA of CA Production by CCD

Data recovered from the CCD matrix were statistically evaluated for the effect of various concentrations of glucose and TCP as well as their interaction on CA production (Table 3). The ANOVA indicates that the overall model design, all individuals, and quadratics are significant model terms, the only exception was the interaction between both tested variables.

**Table 3:** ANOVA of CCD matrix of CA production on the PNR-based medium based supplemented with TCP and glucose by *Trichoderma longibrachiatum* WKA55.

Source		Sum of squares	Degree of freedom	Mean square	*F*	*p*
Model		33693393.6	5	6738678.7	73.7	0.0001 (Significant)
Individual	TCP	894010.6	1	894010.6	9.8	0.0261 (Significant)
	Glucose	1694439.7	1	1694439.7	18.5	0.0077 (Significant)
Interaction (TCP × Glucose)		440696.8	1	440696.8	4.8	0.0796 (Not significant)
Quadratic	(TCP)^2^	21194198.8	1	21194198.8	231.7	<0.0001 (Significant)
	(Glucose)^2^	18449301.4	1	18449301.4	201.7	<0.0001 (Significant)
Residual		457363.7	5	91472.7		
lack-of-fit		437637.1	3	145879.0	14.8	0.0640 (Not significant)
Pure error		19726.6	2	9863.3		
Corrected total		34150757.3	10			
Model evaluation statistics						
Determination coefficient (*R*^2^)			0.9866		
Adjusted-*R*^2^			0.9732		
Predicted-*R*^2^			0.9076		
Adequate precision, %			19.463		
The predicted residual sum of squares			164.20		

Various model evaluation statistics were calculated, of which the lack-of-fit was found to be not significant (*F*-value = 14.8 and value of *p* = 0.0640). The statistical analysis revealed, also, elevated rates of the coefficient of determination *R*^2^ (0.9866) and adjusted-*R*^2^ (0.9732), similarly, predicted *R*^2^ recorded a high value, being 0.9076. The adequate precision ratio was high and measured to be 19.463. The predicted residual sum of squares (PRESS) recorded a reasonably small value of 164.20.

#### Contour Plot and Model Evaluation

**Figure 3:**
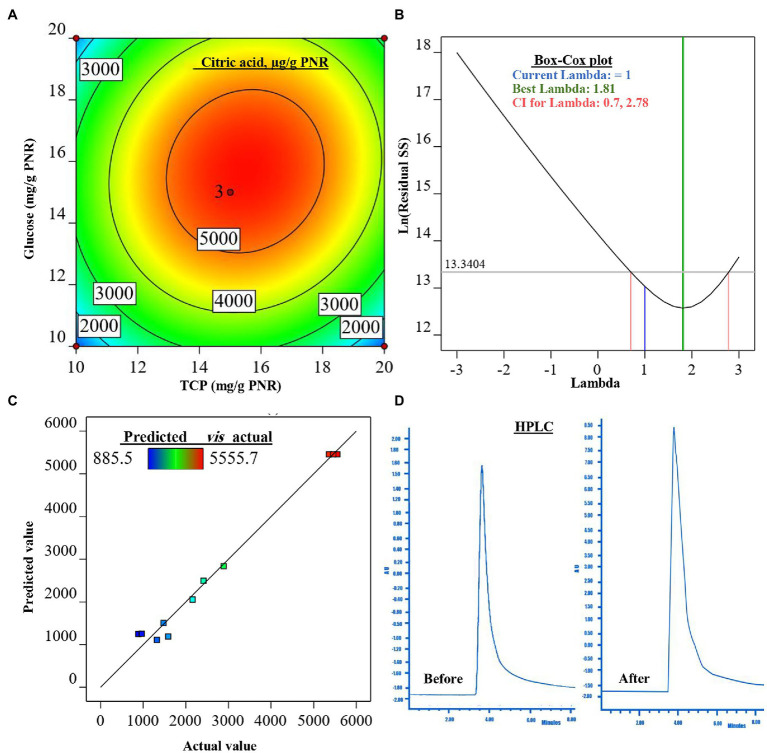
The figure shows the contour plot of citric acid (CA; μg/g PNR) biosynthesis by *Trichoderma* sp. WKA55 as a response to the combination of various concentrations of glucose and TCP on a PNR-based medium **(A)**, the plot of Box-Cox for power transforms **(B)**, predicted versus actual data of CA **(C)**, and **(D)** the diagram of the detected CA by HPLC, before and after the optimization process.

The surface plot of CA was constructed in the form of a contour graph to explore the association between the two tested input variables (glucose and TCP) and the CA response (Figure 3A). As is shown, the continual increase in CA biosynthesis was noted when both concentrations of glucose and TCP amplified, reaching the maximum of CA at the center points (middle concentrations) of both variables, then declined with the higher concentrations afterward.

For model evaluation, the validity checking tests were performed to verify the cogency of the attained polynomial model and detect a value, or group of values, that are not easily predicted by the model. The graph of the Box-Cox plot (Figure 3B) declares that the current lambda (*λ*) was equal to 1.0, the best *λ* was 1.81, whereas the CI was between low (0.70) and high (2.78). In consent with the Box-Cox plot, the plot of actual values versus the estimated (predicted) response values of CA was generated (Figure 3C). As could be seen all data points scatter symmetrically and fragmented evenly along the 45-degree line.

#### Modeling of CA Production

The preceding statistical tools approved and confirmed a satisfactory adjustment of the quadratic model to the investigational data. This model was used to estimate the optimum level of each tested variable (glucose and TCP), as well as the estimated amount of *CA*. The equation that describes the theoretical relationship between the two tested variables that maximize CA production in actual units was generated in terms of the second-order polynomial model to be:


(5) $$\begin{array}{c} CA\;\left(\mathrm{\mu g}/g\;PNR\right)=-27642.09+2061.89\\ \times \mathrm{glucose}+2192.47\times TCP+13.28\times \mathrm{glucose}\\ \times TCP-72.30\times {\mathrm{Glucose}}^{2}-77.49\times {TCP}^{2} \end{array}$$

The mathematical solution of the previous equation led to the theoretical maximum value of CA = 5505.1 μg/g PNR, this could be achieved when fermenting 1 g of PNR with 15.683 mg glucose and 15.490 mg TCP. To confirm the aptness of this mathematical hypothesis, these theoretical concentrations were laboratory validated at the same fermentation conditions. The actually-resulted yield of CA at the laboratory was found to be 5515.9 μg/g PNR. The diagram (Figure 3D) of the HPLC analysis of CA before (898.1 μg/g PNR) and after optimization was introduced, showing a 6.14-fold increase in CA biosynthesis.

### Evaluation of Crude CA

#### CA Against Mycotoxinogenic Fungi

**Figure 4:**
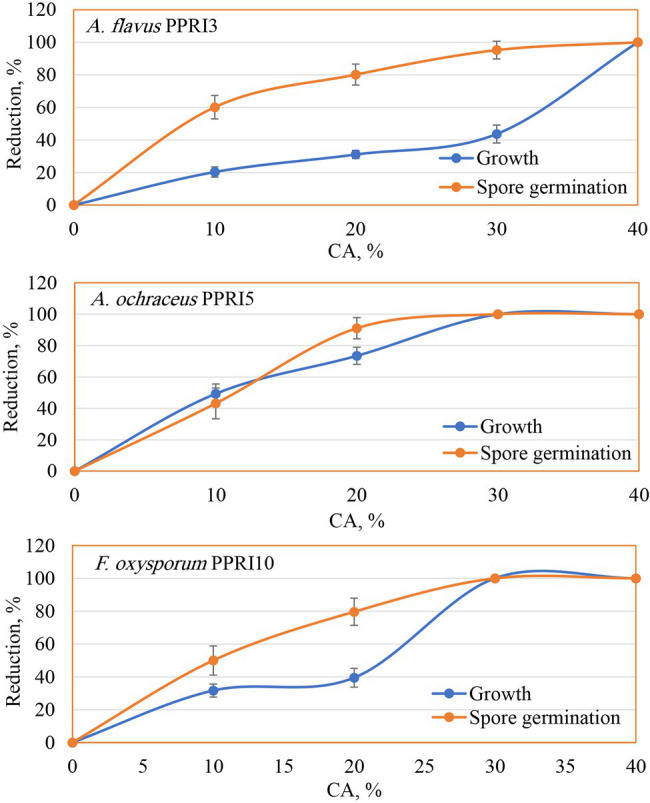
Reduction of growth and spore germination of toxinogenic fungi as affected by various crude CA concentrations.

The biological potential of CA was evaluated against the growth and spore germination of three toxinogenic fungi (*A. flavus*, *A. ochraceus*, and *F. oxysporum*). Data (Figure 4) of the MIC show the inhibitory impact on the growth of the tested fungi as the action of CA, even at the lowest tested level (10%) as compared to the controls (0%). Generally, the inhibitory effect was raised with higher concentrations. In this respect, CA at 20% reduced the growth of *A. flavus* down to 31.09%, *A. ochraceus* to 73.51%, and *F. oxysporum* to 39.46%. By increasing the CA concentrations up to 30%, *A. flavus* growth was reduced to 43.67%, and a complete reduction of mycelial growth was observed against *A. ochraceus* and *F. oxysporum*. Spore germination was found to have the same reduction pattern, in which a significant reduction in spore germination of the tested fungi due to CA was noticed, even in the lowest concentrations (10%). Higher concentrations up to 30%, strongly reduced the spore germination of *A. flavus* (95.22%) and inhibited the spore germination of *A. ochraceus* and *F. oxysporum* as compared to the control’s treatments. At 40%, the fungicidal activity appeared against growth and spore germination of all tested fungi.

#### Seed-Borne Fungi Associated With Peanut *vis* CA

The effect of CA on the frequency of seed-borne fungi on peanuts is evaluated (Table 4). Thirteen fungal species belonging to five genera were recovered from the tested samples. *A. niger*, *A. flavus*, *Penicillium* spp., and *Rhizopus stolonifer* were the most abundant, being 28.7%, 21.94%, 20.71%, and 19.22%, respectively. *Fusarium oxysporum* was the most dominant species among all *Fusarium* spp. (8.94%), followed by *F. solani* (4.39%), whereas *F. incarnatum* and *F. verticillioides* were the least dominant (2.79% and 1.48%, respectively). The data declared the diverse effect of CA on the frequency of seed-borne pathogenic fungi of peanut. In this respect, treated seeds by each of CA or the fungicide led to a significant decrease of *A. flavus*, *F. incarnatum*, and *R. stolonifera*. On the other side, both CA and chemical treatments led to the complete absence of *A. ochraceus*, *A. terres*, *F. oxysporum*, and *F. solani*. On the contrary, the frequency of *A. tamarii* and *Penicillium* spp. was not affected by any of the tested CA or chemical treatments.

**Table 4:** Mean initial values (%) of the incidence of fungi in peanut seeds as a response to CA treatment.

Fungus	Control	CA	Fungicide
*Aspergillus flavus*	21.94^a^	5.00^a,b^	1.670^b^
*Aspergillus niger*	28.70^a^	10.72^b^	5.470^c^
*Aspergillus ochraceus*	2.340^a^	0.00^b^	0.00^b^
*Aspergillus tamarii*	2.280^a^	1.67^a^	0.00^a^
*Aspergillus terres*	6.670^a^	0.00^b^	0.00^b^
*Fusarium incarnatum*	2.790^a^	0.72^b^	0.17^b^
*Fusarium oxysporum*	8.940^a^	0.00^b^	0.00^b^
*Fusarium solani*	4.390^a^	0.00^b^	0.00^b^
*Fusarium verticillioides*	1.480^a^	0.00^b^	0.00^b^
*Penicillium* spp.	20.71^a^	7.64^a^	2.570^a^
*Phoma* sp.	1.110^a^	0.00^a^	0.00^a^
*Rhizopus stolonifer*	19.22^a^	4.67^b^	0.830^b^

Different letter(s) within a raw indicates significant differences.

#### Crude CA *Trichoderma* sp. WKA55 CA *vis* Germination

**Figure 5:**
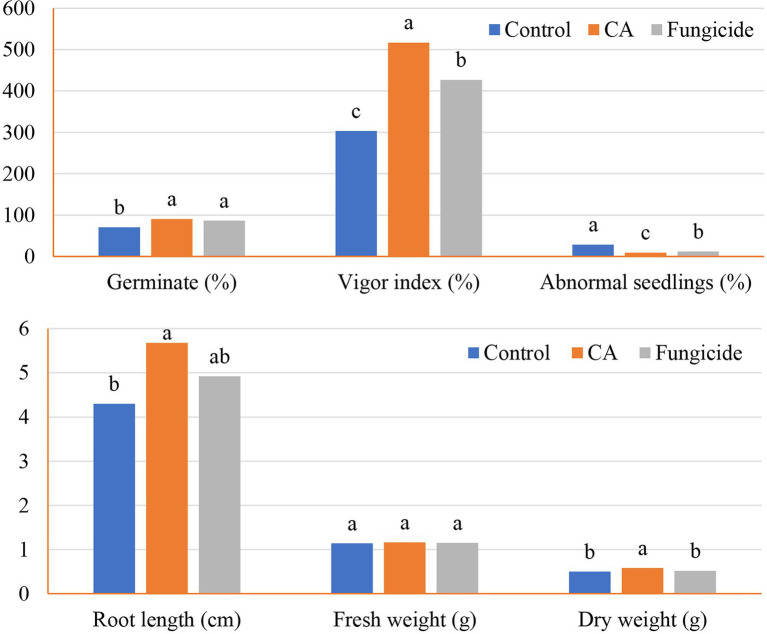
Germination and seedlings characteristics of the tested peanut seeds in accordance with CA treatment.

**Figure 6:**
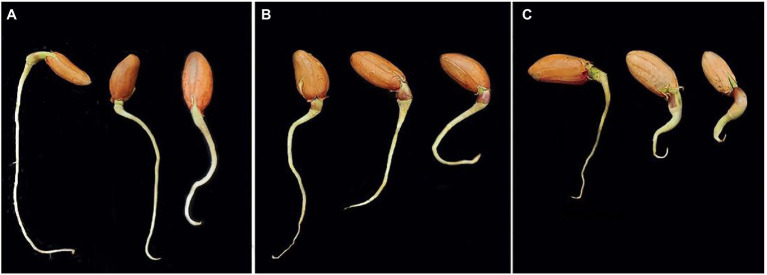
Response of the germinated peanut seeds to various treatments after 14 days. Where, CA treatment **(A)**, fungicide treatment **(B)**, and controls **(C)**, showing the various promoting responses.

The influence of crude CA on the germination of peanut seeds and seedling parameters was also evaluated (Figures 5, 6A–C). The CA was effective in improving the germination percentage (91%) with no significant difference with the chemical fungicide (85%) as compared to the control treatment (70.52%). Compared with the untreated control, seed treatment with each CA and chemical fungicide showed a significant decrease in the percentages of abnormal seedlings. The vigor index was also affected by the application of CA, which increased the vigor index in comparison with the control and showed a comparable trend as in the other parameters. Seeds treated with CA showed a 70.45% vigor index compared with the control of peanut seedlings. Chemical seed treatment came in the second rank, recording 40.61%. Regarding seedling features, the positive effect of the tested CA treatment extended to the root length and dry weight characters of the peanut seedlings, being 36.28% and 16%, respectively, in comparison with the control. On the other hand, no significant variation between CA, chemical fungicide, and control in their effect on fresh weight character.

### GC-MS Analysis of *Trichoderma* sp. WKA55 Crude Extract

**Figure 7:**
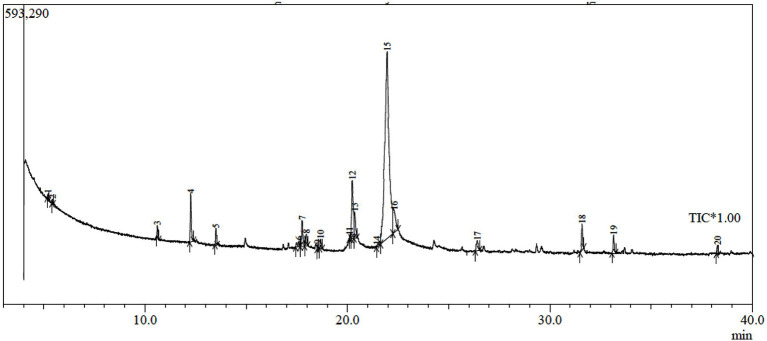
GC-MS chromatogram of fermented peanut plant residual (PNR) by the endophytic *Trichoderma* sp. WKA55, displaying the peaks of various components.

In order to explore the other secondary metabolites in the fungal filtrate produced by the endophytic *Trichoderma* sp. WKA55, the GC-MS was performed (Table 5; Figure 7). A total of 20 unique compounds were detected based on mass spectral properties with area sum % less than 0.05% and up to 65.04% and retention time 5.2 and 33.16 min. These molecules fell into classes of acids, aldehydes, alcohols, ketones, terpenoids, pyrones (lactones), and furanes. The major components in the endophytic filtrates of WKA55 strain were phenol, 2-methyl-5-(1-methylethyl)- (65.04%), followed by benzaldehyde, 4-(1-methylethyl)- (10.37%), 1,3,8-p-menthatriene (8.60%) and 2-cyclohexen-1-one, 2-methyl-5-(1-methylethenyl)-, and (R)- (4.60%). Also, caryophyllene oxide (2.79%), 1,4-cyclohexadiene, 1-methyl-4-(1-methylethyl)- (2.21%), cyclohexanol, 5-methyl-2-(1-methylethyl)-, (1.alpha.,2.beta.,5.al) (1.72%), bicyclo[3.1.0]hexane, 4-methylene-1-(1-methylethyl)-(1.65%), 1-naphthalenol, 1,2,3,4,4a,7,8,8a-octahydro-1,6-dimethyl-4-(1-methylethyl)-(1.33%), 3-cyclohexen-1-ol, 4-methyl-1-(1-methylethyl)-(1.32%), and propanoic acid, 2-oxo-, ethyl ester (1.12%) were found in moderate levels, while sorbic acid vinyl ester (0.98%), 1,3,6,10-dodecatetraene, 3,7,11-trimethyl-, (Z,E)- (0.81%), methyl alcohol (0.48%), and 1-triazene, 3,3-dimethyl-1-phenyl- (0.25%) were found to be the minor metabolites.

**Table 5:** GC-MS analysis of the endophytic *Trichoderma* sp. WKA55 filtrate, sowing the various compounds detected in the PNR hydrolysate.

Peak	RT	Name	RI	Formula	Area	Area sum %	MW
1	5.195	Methyl alcohol	0	CH_4_O	8,584	0.477	32
2	5.427	Propanoic acid, 2-oxo-, ethyl ester	822	C_5_H_8_O_3_	20,162	1.120	116
3	10.607	Bicyclo[3.1.0]hexane, 4-methylene-1-(1-methylethyl)-	897	C_10_H_16_	29,735	1.651	136
4	12.259	1,3,8-p-Menthatriene	1,029	C_10_H_14_	154,791	8.596	134
5	13.507	1,4-Cyclohexadiene, 1-methyl-4-(1-methylethyl)-	998	C_10_H_16_	39,873	2.214	136
6	17.599	Sorbic acid vinyl ester	990	C_8_H_10_O_2_	17,422	0.968	138
7	17.758	Cyclohexanol, 5-methyl-2-(1-methylethyl)-, (1.alpha.,2.beta.,5.alpha.)-	1,164	C_10_H_20_O	30,883	1.715	156
8	17.964	3-Cyclohexen-1-ol, 4-methyl-1-(1-methylethyl)-	1,137	C_10_H_18_O	23,848	1.324	154
9	*	Benzeneacetic acid, 2-methoxy-, methyl ester	1,349	C_10_H_12_O_3_	*	*	180
10	*	Estragole	1,172	C_10_H_12_O	*	*	148
11	*	Isoflurane	252	C_3_H_2_ClF_5_O	*	*	184
12	20.247	Benzaldehyde, 4-(1-methylethyl)-	1,230	C_10_H_12_O	186,796	10.373	148
13	20.365	2-Cyclohexen-1-one, 2-methyl-5-(1-methylethenyl)-, (R)-	1,190	C_10_H_14_O	82,803	4.598	150
14	21.308	1-Triazene, 3,3-dimethyl-1-phenyl-	1,112	C_8_H_11_N_3_	4,511	0.251	149
15	21.963	Phenol, 2-methyl-5-(1-methylethyl)-	1,262	C_10_H_14_O	1,171,140	65.038	150
16	*	Phenol, 2-ethyl-4,5-dimethyl-	1,340	C_10_H_14_O	*	*	150
17	26.401	1,3,6,10-Dodecatetraene, 3,7,11-trimethyl-, (Z,E)-	1,458	C_15_H_24_	14,573	0.809	204
18	31.587	Caryophyllene oxide	1,507	C_15_H_24_O	50,159	2.786	220
19	33.156	1-Naphthalenol, 1,2,3,4,4a,7,8,8a-octahydro-1,6-dimethyl-4-(1-methylethyl)-,	1,580	C_15_H_26_O	23,912	1.328	222
20	*	Pentanal, 2,4-dimethyl-	777	C_7_H_14_O	*	*	114

RT, retention time; *, the compound detected at less than 0.05%; RI, retention index; and MW, molecular weight.

### Molecular Identification of *Trichoderma* sp. WKA55

**Figure 8:**
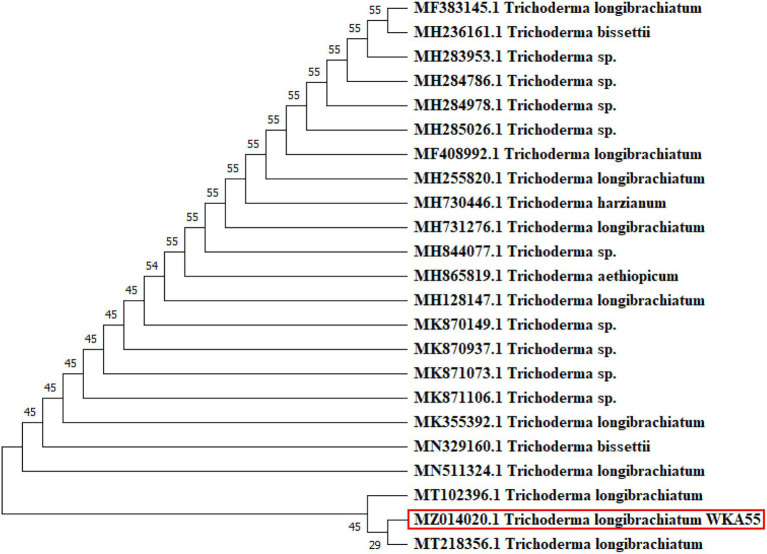
Molecular phylogenetic tree of the sequence of 18s rRNA, showing the position of *Trichoderma* sp. strain WKA55 (surrounded with the red rectangle) within the closely related sequences in GenBank.

Based on the preceding trials, the isolated fungal strain (*Trichoderma* sp. WKA55) was molecularly identified based on 18 s rRNA, as a perfect identification tool. From BLAST results, strain WKA55 displayed high similarity, up to 99.65%, with the previously identified *T. longibrachiatum* strains (MT218356.1, MT102396.1, and MN511324.1) on the GenBank. The constructed phylogenetic tree of *Trichoderma* sp. WKA55 was depicted (Figure 8), which comes in line with the previous morphological identification. The GenBank accession number of the present fungal strain (*T. longibrachiatum* WKA55) was MZ014020.1.

## Discussion

In the current study, a survey of fungal endophytes in of the peanut seeds was managed. The survey yielded 26 endophytic species belonging to 11 genera, of which *Trichoderma* spp., an endophyte bioagent was recorded at moderate percentages (8.60%).

Most of the previous endophytic fungi are isolated from parts other than seeds, and little is known about the endosymbionts of seeds. Most of the endophytic isolates belong to Ascomycota and Deuteromycetes. Previously, 72 species belonging to 21 genera of endophytic fungi were isolated from the roots of three leguminous plants (peanut, alfalfa, and broad bean). In this work, *Fusarium*, *Penicillium*, and *Aspergillus* were the commonly isolated genera, of which *F. solani*, *F. subglutinans*, *F. oxysporum*, *P. duclauxii*, *P. funiculosum*, *A. tubingensis,* and *A. flavus* were the most prevalent (El-Maghraby et al., 2013). Similarly, 17 and 16 species belonging to 11 genera were isolated from common bean and cowpea seeds, respectively (El-Samawaty et al., 2014). However, these studies did not attain fungal strains with significant features. On the other side, isolation of diverse fungal genera from peanut seeds in the current investigation may be back to the nutrient composition of such pulses, which resemble a nutritious substrate for fungal growth and spore establishment (El-Samawaty et al., 2014). The high incidence of some pathogenic fungal genera in peanut seeds may be back to the aggressivity of these pathogens in the field, and later under improper storage conditions play a life-threatening role in humans and cause several damages in pulses as well (Santos et al., 2016; El-Zefzaf, 2020).

The current study reported endophytic fungi with lytic activity on plant biomass residuals. The biodegradation of such residuals comprises the first step in the bioconversion process. Earlier, two *Aspergillus* spp., two *Penicillium* spp., and one *Trichoderma* sp. were isolated from rice straw and showed high cellulase activity (Saber et al., 2010). Fungal ability to decompose the crystalline cellulosic biomass depends upon the presence of complementary cellulases profiles (endoglucanase, exo-glucanase, and β-glucosidase) in sufficient amounts (Al-Askar et al., 2021b). The fungal cellulolytic activity could be considered indicators of the degradation of PNR by the tested fungi, leading to the release of pentoses and hexoses monomers as the main units, representing the cornerstone for the formation of various organic molecules, including organic acids.

Seven selected endophytic fungi were further investigated for cellulosic activity and complex phosphate solubilization ability. The released glucose as a function of cellulase activity in the present work is lower than stated earlier, suggesting the speedy and/or direct biotransformation of the liberated glucose into biomolecules such as value-added organic acids. This type of bioprocessing that accompanied by low free glucose in the fermentation medium could be considered the initial indicator for the direction of production of valuable compounds. FPase is the overall cellulolytic activity and plays a critical role in the bioconversion of lignocellulosic into fermentable sugars (Saber et al., 2015; Elsayed et al., 2021). Accordingly, this study could be considered a pioneer regarding the screening of the lytic enzymes for the selection of endophytic fungus. What is more, these fungi could be a new source of enzymes with wide biotechnological applications.

The maximum liberation of free P was achieved by *Trichoderma* sp. WKA55 (15.37 μg/g PNR), confirming the competence of such isolate as a potent solubilizer of complex phosphate. Several filamentous fungi, mainly black Aspergilli, some species of *Penicillium*, and rarely *Trichoderma* spp. were reported as complex phosphate solubilizers, the solubilization of complex phosphate and release soluble P is usually linked with the generation of organic acids, reduction of medium pH, and/or production of various organic molecules (Max et al., 2010; Show et al., 2015; Elsayed et al., 2021).

Both cellulases (FPase and CMCase) of *Trichoderma* sp. WKA55 showed obvious activity during the 15 days of fermentation. This, in turn, indicates that both enzymes are induced and could be also constitutive, that is to say, it could be found associated with the fungus, even in the absence of their inducer substrate, also indicate that *Trichoderma* sp. WKA55 has a complete cellulolytic system that can completely degrade the majority of PNR cellulose. In this connection, hydrolysis of PNR cellulose requires (i) endoglucanase (1,4-β-D-glucan-4-glucanohydrolases, EC 3.2.1.4) that randomly cleaves the internal β-1,4-glucosidic bonds, (ii) exoglucanases (1,4-β-D-glucan glucanohydrolases, EC 3.2.1.74 and 1,4-β-D-glucan cellobiohydrolases, EC 3.2.1.91), which act on the reducing or nonreducing terminals of cellulose chains, liberating glucose and/or cellobiose, exoglucanases can also catalyze the peeling of the microcrystalline structure on cellulose chains, and (iii) β-glucosidase (β-glucoside glucohydrolases, EC 3.2.1.21) that releases glucose monomers from cellodextrins and cellobiose (Saber et al., 2015; Elsayed et al., 2021).

Marked xylanase activity along the 15 days incubation period was noticed by *Trichoderma* sp. WKA55, suggesting the ability of such fungus to catalyze xylan of hemicellulose part of PNR into xylose monomers. The continual secretion of such xylanase along the fermentation period is an indication of the induced and constitutive nature of the enzyme. The complete hydrolysis of xylan begins with endoxylanase (1, 4-β-D-xylan xylanohydrolase; EC 3.2.1.8), which cleaves the internal glycosidic bonds, accumulating xylooligomers of β-D-xylopyranosyl that may inhibit the endoxylanase, then xylosidases (1,4-β-D-xylan xylohydrolase; EC 3.2.1.37), hydrolyzes the xylooligomers, thus removing the cause of inhibition and releasing D-xylose from the xylooligomers; finally, acetylxylan esterase (EC 3.1.1.6) eliminates the O-acetyl groups from positions 2 and/or 3 on the β-D-xylopyranosyl residues (Abdelwahed et al., 2011; Elsayed et al., 2021).

Several kinds of proteases (such as peptidyl/peptide hydrolases, EC 3.4.21-24, and 99) can degrade nitrogenous complexes, and hydrolysis the peptide bonds in a protein fragment (Negm et al., 2021). For a long time, the function of proteases was solely dedicated to protein digestion as a feeding source for the microorganism. Next, it becomes clear that the catalytic action of protease can have a wide range of very specific functions, besides their critical role in the pathogenesis. Six types of proteases were classified based on the catalytic activity, i.e., cysteine-proteases, threonine-proteases, glumatic-proteases, aspartic-proteases, metalloproteases, and serine-proteases, the latter three are the most abundant protease types (Barrett et al., 2012).

Pectin is another complex polysaccharide (composed of α-1, 4-linked D-galacturonic acid) located mainly in the middle lamella, representing the intercellular cement, and helping to bind cells together. Also, in the cell wall pectin forms an amorphous gel, filling the spaces between the cellulose microfibrils (Jacob et al., 2008). Polygalacturonases (EC 3.2.1.15) degrade pectic substances and split the pectin chain by adding a water molecule and breaking the linkage between two galacturonan molecules. Pectin lyases (EC 4.2.2.2) are another enzyme that split the chain by removing a molecule of water from the linkage, releasing unsaturated double-bond products. The synergistic action of both enzymes breaks the pectin chain and releases a single unit of galacturonan (Sathiyaraj et al., 2011), leading to weakening of the cell walls and tissue maceration (liquefaction) thus development of many diseases, particularly those characterized by soft-rotting of tissues. This enzyme system is involved in many phytopathogens that invade the plant tissue (Rahman and Punja, 2005; Al-Askar et al., 2021a).

PNR did not receive any pretreatment before the fermentation, suggesting that *Trichoderma* sp. WKA55 potentially has complete lytic activities (cellulolytic, xylanolytic, proteolytic, and pectinolytic), which enabled the fungus to penetrate and degrade the complex structure of PNR. Reduction in residual PNR biomass after fermentation could be considered a shred of evidence for the transformation of the fermented PNR into other organic molecules. Another, the scenario of pH during fermentation is very important, especially for CA production, since spore germination requires pH higher than 5; then, the absorption of ammonia in the medium by germinating spores causes the release of protons, this, is in turn, lowers the pH and improves the generation of CA, and the low pH value during the fermentation process inhibits the production of unwanted organic acids (Max et al., 2010).

It was better to manage the minimization of the bioconversion process into one direst step by supplementing the fermentation medium with a complex phosphate, e.g., tricalcium phosphate, as a sole phosphorus source. This urged the microorganism to subtilize complex phosphate for getting the free phosphorus required for various physiological activities in microbial cells, such as the formation of nucleic acids and the generation of ATP. This directed microbial solubilization of complex phosphate is usually convoyed by and attributed to the formation of organic acids, which are also stated as the end-molecules of microbial decomposition of cellulose (Kumari et al., 2008; Al-Askar et al., 2021b). That is why cellulose-degrading enzymes and complex phosphate are prerequested in the organic acid production medium.

Three microbial mechanisms were reported for solubilization of complex phosphate; (1) the catalytic action of phytase and/or acid phosphatase, (2) the secretion of organic acids; mainly citric, oxalic, and succinic acids, and/or (3) the drop in pH (Saber et al., 2010; Elsayed et al., 2021). It has been stated that the gradual reduction in pH during biofermentation led to the formation of organic acids; therefore, the presence of complex phosphate as the only source of P in SSF medium induced the fungus toward the assimilation, solubilization, and utilization of complex phosphate, by the secretion of enzymes (phytase and/or acid phosphatase) and/or the production of organic acids (Kumari et al., 2008; Max et al., 2010; Elsayed et al., 2021), the latter is in charge for lowering the pH.

The hydrolytic enzymes reached their maximum on the 11th day of fermentation, but the production of organic acids was maximized on the 9th day so, 9 day was considered as the chosen biofermentation period. However, this period is relatively accepted in relation to the expected valuable products, and further, is comparable with 28 days fermentation for biodegradation of wheat straw and cellulase production under SSF (Dinis et al., 2009) and 4 weeks on wheat and rice straws (Saber et al., 2010).

However, plant biomass residuals, such as PNR, are a suitable raw material for fermentation only if the microorganism has the enzymatic system required for fermentation and catalyze effectively at low pH values (Max et al., 2010), this is what exactly happened by the present *Trichoderma* sp. WKA55. Nevertheless, the previous tests could conclude two approaches: one of them is an indirect, represented by the cellulolytic activity, reduction of residual PNR biomass, and solubilization of TCP by the tested fungal strain; the other direct approach is the biosynthesis of organic acids.

The current endophytic fungus (*Trichoderma* sp. WKA55) might be a novel source of hydrolytic enzymes of broad biotechnological uses. Obtaining fungal strain that has a complementary degradation system of plant biomass residuals and, at the same time, solubilizing the complex phosphate could be managed genetically, but isolation from the native environment may be better for adaptation and succession when incubated with a medium containing plant biomass residual. Because of *Trichoderma* sp. WKA55 has met these hypotheses, it was chosen for identification based on the morphological structures of vegetative mycelia and light microscope investigation, in addition to the molecular identification.

Perfectly, the present candidate fungus was proved to have the complete and complementary catalytic action to manage the transformation of PNR into other molecules, i.e., organic acids. A primary test using HPLC declares CA as the major detected organic acid in the fungal filtrate. Therefore, an optimization trial using CCD was performed, taking into account the maximization of CA biosynthesis using PNR as a fermentation substrate.

The CCD of RSM procedure generates the maximum amount of knowledge from the minimum number of experiments. This is an important issue, considering the cost of HPLC analysis. In contrast to the one-variable-at-a-time method, RSM can find the true optimum conditions, and interactions of the bioprocess would have been made available. What is more, all the experimental trials are carried out simultaneously; thus, the results are obtained rapidly (Saber et al., 2015).

Once applying the CCD, it was found to support the fermentation medium with a low level of glucose to support the biosynthesis of *CA*. As it is already known that glucose has a stimulatory action on CA production (Max et al., 2010). Besides reducing the overall process cost, the application of RSM in biofermentation technology maximizes the target yields. Herein, PNR was applied as one support-substrate phase, i.e., solid medium support for SSF, and at the same time, carbon and nitrogen sources for CA production. This is expected to be friendly for both coasts and the environment. The highest value of CA has located nearby the design center points of the examined variables, returning the precision and fitness of the selected concentrations of both variables.

The significance of model terms through ANOVA indicates the importance of the parameters in the experimental design for CA production. Model evaluation statistics were calculated to assess the model, the lack-of-fit was calculated to give information about the suitability of the chosen variables, consequently, the appropriateness of the obtained data to create a new model.

The regression model exhibits a significant lack-of-fit when it fails to designate the functional relationship between the experimental input factors and the response output variable(s). Lack-of-fit can occur if individuals, interactions, and/or quadratic terms from the model are not included in the final model. It can also occur if several unusually large residuals resulted when fitting the model. Fortunately, the present lack-of-fit of the model was not significant; therefore, the model is fitted and reflects a good parameter.

*R*^2^ is a diagnostic tool, that ranges from 0 to 1, that measures the exactness of the model, the higher value, the greater the association between the variables and target response (CA). High values of the three kinds of *R*^2^ in the current work indicate a high relationship between the experimental parameters (glucose and TCP) and the response (CA). Similarly, high predicted *R*^2^ indicates how the high accuracy of the model to forecast the new observations, the higher the predicted *R*^2^ value, the greater the predictive capability of the model. Accordingly, the current model could help in the prediction and estimation of CA from any given concentration from glucose and TCP with 90.76% accuracy.

The adequate precision that measures signal-to-noise ratio was found to be 19.463; however, a value greater than 4 is desired. The high ratio of the present model indicates an adequate signal. Thus, the models can be used to predict the values of responses and can be used to predict CA values along with the design space; this is a critical requirement for a model together with the non-significant lack-of-fit, as the fitting of the model is required.

PRESS is another statistical parameter that is usually used for evaluating the model and calculation of the predicted *R*^2^. The lesser the PRESS value, the superior the model’s forecasting capability. Both predicted *R*^2^ and PRESS minimize the overfitting of the model (misleading regression coefficients) by preventing random error or noise.

The contour plot showed the maximum of CA at the middle concentrations of both glucose and TCP, then declined with the higher concentrations; this, in turn, indicates the accuracy of the tested variables and their concentrations as well.

Regarding the validity checking tests, the Box-Cox plot for power transforms displayed the best value of *λ* value between the points of low and high C.I., indicating that the current model predicts and reflects efficiently the real system and thus, there was no need for data transformation. Plotting the actual vis, the predicted values showed that the data points divided evenly along the 45-degree line, indicating that all values can be easily predicted by the model. Such a scattering pattern around a 45-degree diagonal line approves an acceptable correlation between the predictive and experimental values of *CA*. This is the ideal distribution of the data points, which fulfill the optimum fitness of the model. This, in turn, indicates that all run points could be precisely predicted by the model.

Laboratory validation of the fermentation conditions, based on the CCD modeling process, revealed that the variance between the theoretical (5505.10) and experimental (5,515 ± 21) values of CA is small enough (10.11 μg/g PNR) to fortify the fitness of the model. However, such medium constitution is characterized by simplicity and economy, in relation to the high valued-added CA produced under SSF of PNR by *Trichoderma* sp. WKA55.

During this study, the resulting CA in the hydrolysate of SSF by the cellulolytic, proteolytic, and TCP-solubilizing *Trichoderma* sp. WKA55 was quantified using HPLC. Because of speed, simplicity, and accuracy, the HPLC technique is an appealing procedure for such detection. CA could be produced through SSF and submerged fermentation. Here, the SSF technique was applied to get the advantages of simplicity, low energy requirements, high volumetric productivity, ease of aeration, and simulation of the natural habitat of most fungi. CA was reported to be delivered by several fungi, involving *Aspergillus* spp. and *Penicillium* spp. (Max et al., 2010; Saber et al., 2015), but little is known about its production by the current endophytic strain; *Trichoderma* sp. WKA55, representing a new source for CA production. Hydrolysis, biochemical metabolism, and microbial assimilation are suggested mechanisms for the formation of organic acids in the fermented matter. Likewise, all organic acids, CA has its biosynthesis pathway, it was found to be promoted as the major acidic metabolite of *Penicillium bilaii* under nitrogen-limited conditions, in some cases CA share the same pathway of oxalic acid formation, and both are at one enzymatic step from the main metabolism of D-glucose and D-fructose pathway (Max et al., 2010; Show et al., 2015; Al-Askar et al., 2021b). By the end of optimization trials, a total yield of 5,515 ± 21 μg CA/g PNR was attained, representing a new pioneer fungal candidate for CA biosynthesis. The current yield is higher than (2.007 μg/g) these obtained recently on maize stover (Al-Askar et al., 2021b).

The biological potential of CA against some important toxicogenic fungi showed a marked reduction in growth and spore germination against the toxinogenic fungi to prevent or at least reduce their activities. Because of their direct hazards and devastating influence on human health, diseases produced by mycotoxigenic fungus have received special attention. Their mycotoxins are widespread, and they are produced under a wide range of environmental circumstances (Bennett and Klich, 2003; Marin et al., 2013). Individuals of the genus *Fusarium* and *Aspergillus* were reported to produce groups of mycotoxins, contamination of agricultural products by such toxigenic and pathogenic fungi plays a crucial role in terms of economy, hygiene, and health. Among mycotoxins, aflatoxin, fumonisin, and ochratoxin are the more toxic to organisms, causing an assortment of dangerous impacts including teratogenicity, hepatotoxicity, and mutagenicity (Marin et al., 2013). Mycotoxins secreted by such fungi devastate the yields during production, storage, processing, and even in the markets, leading to losing their nutritious value (Jimoh and Kolapo, 2008).

The utilization of fabricated fungicides for the elimination of toxinogenic fungi tends to the development of various known perils. Alternative bio-fabricated ones have been proposed. In this regard, the mycotoxin content was reduced after treatment with the fungal filtrate, and more, the antimicrobial property of CA against a vast array of microorganisms was demonstrated. For instance, in an *in vitro* study, great inhibition was noticed on the linear growth, dry weight, and sporulation of the soil-borne pathogens; *F. solani* FP2 and *F. oxysporum* FP4 by the action of CA (Abdel-Monaim and Ismail, 2010). Similarly, a significant growth reduction of wheat seed-borne pathogens (*F. culmorum*, *F. moniliforme*, and *F. graminearum*) grown on media amended with different concentrations of CA (Seadh and El-Metwally, 2015). In the two investigations, the reduction in fungal growth was linked to the increase in CA levels.

The antifungal action of CA could be ascribed to several pathways such as the decline of internal pH of the microbial cell by ionization of undissociated acid molecules and disturbance of system transportation by changing the permeability of cell membrane and/or reduction of proton motive power (Shokri, 2011). In addition, some antifungal metabolites in the culture filtrate may synergistically have contributed to the antifungal nature of *Trichoderma* sp. WKA55 filtrate. Moreover, the evolution of the endophytic fungi to elaborate many novel metabolites may be back to their multiple interactions inside the host plant, i.e., with the plant tissue, or with the endemic pathogens, or even among each other (Schulz et al., 2015). The antifungal role of the lytic activity of *Trichoderma* species was reported against the growth of *Rhizoctonia solani* pathogen. The isolate can produce pectinase and chitinase and solubilize phosphorus (Sallam et al., 2021).

As a practical applicable approach, peanut seed treatment by the filtrate of *Trichoderma* sp. WKA55 led to a marked improvement in reducing the fungal load on the seed. Such data encourage the application of the current *Trichoderma* filtrate as pretreatment to minimize the fungal load of peanut seeds, especially the toxicogenic ones. However, the current data have another advantage from the nutritional point of view, since the mycotoxins contamination caused by mycotoxinogenic fungi is a great concern in nuts production worldwide.

Several studies stated that metabolites produced by *Trichoderma* spp. had a positive promoted plant growth without negative impact on the plants. In the present study, there was a positive effect of crude CA on seed germination and seedling features that may be back to the high capacity of *Trichoderma* species to secret plant-growth promotors that act as auxin-like molecules, e.g., gibberellic acid and indole acetic acid, and the secretion of antibiotic-like substances as a biocontrol agent, which possibly act as auxin-like molecules (Vinale et al., 2014; Saber et al., 2017; Singh et al., 2018; Al-Askar et al., 2021b). Recently, a pronounced increase of seed germination, root and shoot lengths, plant dry matter, and vigor index of durum wheat seeds coated with three different strains of *T. harzianum* and the *Trichoderma*-based commercial product; Trianum-T22 were reported (Kthiri et al., 2020). These results were also confirmed on tomato seeds by *T. asperellum* CA, which has the potential to enhance germination percentage, root length, and vigor index parameters up to 91%, 8.85%, and 49.40%, respectively (Al-Askar et al., 2021b). Another critical role of *T. asperellum* was stated to induce H^+^-ATPase, which is an important enzyme responsible for cell growth and plasma membrane elongation during the growth of maize seedlings (López-Coria et al., 2016).

In the present study, secondary metabolites other than CA in the fungal metabolite were explored, through GC-MS, for a possible role of antibiosis and pathogenic activity, a total of 20 compounds from the culture filtrate of the endophytic *T. longibrachiatum* WKA55 strain were determined. Among microorganisms, the secondary metabolites of the genus *Trichoderma* have a wide biological role in agricultural, industrial, and medical aspects. With minimal nutritional requirements, *Trichoderma* species secrete a plethora of metabolites such as terpenes, gliotoxin, pyrones, gliovirin, and peptaibols (Vinale et al., 214; Khan et al., 2020). These secondary metabolites have antimicrobial features, acting as potential biocontrol agents against phytopathogenic antifungal activities (Vizcaino et al., 2005). *Trichoderma longibrachiatum* is one of the recent studied fungal biocontrol agents, to date, several strains have been reported to generate a diversity of new volatile and nonvolatile metabolites, including peptides, polyketides, and terpenes, which led to the appreciation of *T. longibrachiatum* as a potential source of unique antibiotics and as talented biocontrol agents against phytopathogenic microorganisms and nematodes (Sridharan et al., 2020, 2021). These metabolites were found to delay the colonization of the pathogens; for instance, the antifungal secondary metabolites of *T. longibrachiatum* were reported against *Candida albicans* and *Pyricularia oryzae* (Xuan et al., 2014). Further, the secondary metabolites were found to induce disease resistance and regulate plant growth (Khan et al., 2020).

Among active constituents detected in the current fungal filtrate, phenol, 2-methyl-5-(1-methylethyl)-, or carvacrol was found to be the major unique volatile compounds in the tested extract (65.04%), which was, recently, demonstrated as among the main active ingredients of *T. harzianum* filtrate with significant antifungal activity against the phytopathogenic *Rhizoctonia solani* (Yousef et al., 2018). Similarly, phenol 2,4, bis (1,1-dimethyl ethyl) was reported as a main secondary metabolite in the filtrates of *T. harzianum* and *T. asperellum* that prompted the germination and the growth of the tomato seedling (Yuef et al., 2018).

The present fungal strain showed the presence of benzaldehyde, 4-(1-methylethyl)-, caryophyllene oxide, and propanoic acids in the filtrate. These compounds showed antifungal properties that inhibit the mycelial growth of different phytopathogens (Lee, 2015; Yassin et al., 2021). Benzaldehyde, 4-(1-methylethyl)- was reported as a volatile organic compound in *T. atroviride* extract and exhibited very strong antifungal activity at low concentrations (Lee, 2015). Several positive biological benefits of caryophyllene oxide, secreted by *T. viride*, which reduced the growth of *Fusarium solani*, *Rhizoctonia solani*, and *Sclerotium rolfsii* pathogens (Awad et al., 2018). Also, caryophyllene oxide, propanoic acid, and 1 pentanol were reported among active constituents of *T. viride* filtrate, against the growth of *F. verticillioides* and *F. proliferatum* maize fungal pathogens (Yassin et al., 2021).

The antifungal activity of *T. longibrachiatum* metabolites was recently explained against *Sclerotium rolfsii* and *Macrophomina phaseolina*. During the direct *T. longibrachiatum*-pathogen interactions, an alteration and reduction of the mycelial growth and inhibited the production of sclerotia of the pathogens were noticed, which was associated with secretion of several antifungal compounds including longifolene, 1-butanol 2-methyl, cedrene, caryophyllene, and cuprenene, which are involved in the biosynthetic pathways of the sesquiterpenoid and alkane, and the degradation pathway of trimethylamine. What is more, 1-pentanol, 1-hexanol, myristonyl pantothenate, bisabolol, d-Alanine, and diethyl trisulphide draws attention as a plant-growth promoting and unique antimicrobial compounds (Sridharan et al., 2020, 2021).

GC-Mass analysis of the endophytic fungus studied *T. longibrachiatum*_strain WKA55 (MZ014020.1) revealed the presence of several volatile organic compounds (VOCs) belonging to a large class of compounds comprising hydrocarbons, alcohols, ketones, aldehydes, esters, acids, and terpenes, as well as caryophyllene derivative. In this respect, epi-β-caryophyllene was detected among the bioactive VOCs, which have been reported to possess properties of antifungal, and antioxidants activity, as well as promoting seedling growth and chlorophyll content (Lee et al., 2016; Rajani et al., 2021). *Trichoderma* species have been reported as excellent plant-growth-promoting fungi, which can improve plant health by creating a favorable environment and production of a large number of secondary metabolites such as harzianic acid, harzianolde, abscisic acid, auxin-related compounds indole-3-acetic acid, indol-3-acelaldehyde, and indol-3 ethanol (Zin and Badaluddin, 2020). Some others produce valuable VOCs with different mechanisms involved in biological control, inducing defense responses and promoting plant growth (Phoka et al., 2020; Wonglom et al., 2020; Ruangwong et al., 2021). However, the approach of biological actions of the current fungal metabolites are unique and numerous, yet a small portion is known. Collectively, the potential uses of these metabolites include may include the volatile-mediated inhibition of pathogen growth and augmented plant systemic resistance. The antifungal activity cannot be attributed to CA or the main metabolite [phenol, 2-methyl-5-(1-methylethyl)-] only but also to the occurrence of other bioactive constituents, which may attribute synergistically to exert an antifungal power.

Finally, the current endophytic fungal strain (*Trichoderma* sp. WKA55) was identified using the molecular procedure as a perfect means for identification. Because of high sensitivity and specificity, molecular identification is widely used for the rapid identification of filamentous fungi at various taxonomic levels. The technique is set up for the comparison of the sequence coding for 18 s rRNA gene after PCR amplification, through the ITS, which fragment size is uniform in numerous groups of fungi, making nucleotide sequencing of ITS fractions prerequisite for revealing interspecific, and in some cases, intraspecific variation (Al-Askar et al., 2021b). *Trichoderma longibrachiatum* belongs to the clade longibrachiatum, sordariomycetes, order: hypocreales, and family hypocreaceae (Hewedy et al., 2020).

Summing up, a novel endophytic isolate was reported as a biologically active fungus capable to produce CA, through a safe and economic disposal procedure of PNR biomass. The basic concepts for the bioconversion process of PNR into CA, on a simple and low-cost medium using a new endophytic *T. longibrachiatum* WKA55, were illustrated. The unique complementary hydrolytic system of *T. longibrachiatum* WKA55 enables efficient bioconversion of plant biomass without any previous pretreatments, suggesting a novel source of broad biotechnological enzymes as well. The crude fungal exudate was able to suppress the pathogenic and mycotoxin-producing fungi associated with peanut seeds; as well as improve the germination and growth of peanut seedlings. Therefore, large-scale production is very encouraged especially with incremental production of PNR. The current concept may be applied for the breakdown of the infection cycle of the various pathogens that probably exist. It is worthy to note that the seasonal climatic variation may affect the stability of the percentage of the compositional constituents of PNR, which may reflect on the production stability of fungal metabolites. However, this drawback is very limited, and variation is ranging within a narrow range. Nevertheless, the suggested point for further studies could be that the residual fermented PNR matter could be used as a soil amendment or as an animal feed supplement, representing integrated management of such biomass residue. To gain a better understanding of the influence of crude CA produced *via T. longibranchiatum* WKA55 in restricting mycotoxgenic pathogens growth and stimulation the seedlings growth, other work recommended under field conditions to extensively evaluate the obtained compounds on the large scale.

## Data Availability Statement

The datasets presented in this study can be found in online repositories. The names of the repository/repositories and accession number(s) can be found in the article/supplementary material.

## Author Contributions

ER, ZM, KG, and WS contributed to the conception and design of the study. AA, ER, ZM, AM, and FA-O organized the database. AA-A, ER, ZM, AM, and WS performed the statistical analysis. AA-A, ER, ZM, KG, and FA-O wrote the first draft of the manuscript. AA-A, ER, ZM, KG, FA-O, AA, and WS wrote the discussion section. AA and WS substantially contributed to the conception of the work and interpretation of data and critically revised the intellectual content of the manuscript. All authors contributed to manuscript revision, read, and approved the final version.

## Funding

The authors extend their appreciation to the researcher supporting project number (RSP2022R505), King Saud University, Riyadh, Saudi Arabia for funding this work.

## Conflict of Interest

The authors declare that the research was conducted in the absence of any commercial or financial relationships that could be construed as a potential conflict of interest.

## Publisher’s Note

All claims expressed in this article are solely those of the authors and do not necessarily represent those of their affiliated organizations, or those of the publisher, the editors and the reviewers. Any product that may be evaluated in this article, or claim that may be made by its manufacturer, is not guaranteed or endorsed by the publisher.

## Footnotes

^1^ http://www.ncbi.nlm.nih.gov/BLAST

## Abbreviations

PNR: Peanut plant residual
CA: Citric acids
TCP: Tricalcium phosphate
FPase: Filter-paperase
CMCase: Carboxymethyl cellulase
PGase: Polygalacturonases
VOCs: Volatile organic compounds
